# Relation of Mindfulness, Heartfulness and Well-Being in Students during the Coronavirus-Pandemic

**DOI:** 10.1007/s41042-022-00075-1

**Published:** 2022-08-31

**Authors:** Martina Rahe, Fabian Wolff, Petra Jansen

**Affiliations:** 1 University of Koblenz-Landau, Universitaetsstrasse 1, 56070 Koblenz, Germany; 2 University of Regensburg, Universitaetsstrasse 31, 93053 Regensburg, Germany

**Keywords:** Mindfulness, Gratitude, Self-Compassion, Well-Being, Coronavirus Pandemic, Physical Activity

## Abstract

The Coronavirus Pandemic has affected the lives of almost everyone. The study’s primary goal is to analyze how mindfulness and heartfulness (gratitude and self-compassion) predict well-being and flourishing during the pandemic. Participants were 216 students completing questionnaires about well-being, flourishing, mindfulness, self-compassion, gratitude, physical activity, Coronavirus stress and strain, and demographic information. Participants’ physical activity, strain, stress, and gender were also investigated as possible predictors. Mindfulness predicted well-being and flourishing. These relations were mediated by gratitude and self-compassion. Physical activity also predicted well-being and flourishing, but the Coronavirus stress and strain were unrelated to students’ well-being or flourishing. In an exploratory analysis, three aspects of mindfulness (non-judging, observing, and acting with awareness) predicted well-being, and four (non-judging, describing, observing, and acting with awareness) predicted flourishing. Aspects of heartfulness mediated the relations between these aspects of mindfulness and well-being and flourishing.

## Relation of Mindfulness, Heartfulness, and Well-Being in Students during the Coronavirus-Pandemic

Due to the Coronavirus-Pandemic, almost every person’s life has changed, and their subjective well-being has been influenced at some point (Zacher & Rudolph, 2021). In a German sample, life satisfaction decreased during the early stages of the Coronavirus Pandemic between March and May 2020, and personal well-being could be predicted by general life satisfaction and gratitude (Büssing et al., 2020). However, students in higher education were affected by the Coronavirus Pandemic. Universities were mainly closed, and distance learning became the norm for several semesters. 86.8% of students felt that the pandemic significantly influenced their studies, and well-being was low or very low for 65.3% (Dodd et al., 2021). American students’ stress increased during the pandemic, while at the same time, they reported a significant decline in physical activity (Wilson et al., 2021), and sedentary behavior increased (Bertrand et al., 2021). Nevertheless, physical activity and mindfulness were protective against stress in the pandemic: The time spent with physical activity was related to positive affect before and during the stay-at-home orders (Maher et al., 2021). Also, mindfulness-based stress-reduction programs can improve mental well-being and emotional regulation during the Coronavirus pandemic (Sanilevici et al., 2021). In general, dispositional mindfulness is negatively linked to anxiety, stress, and depression (Sharma & Kumra, 2022). Beside this, heartfulness is related to well-being (Voci et al., 2019). According to this, it is the primary goal of this study to investigate if there is also a relation between dispositional mindfulness, dispositional heartfulness and well-being during the Coronavirus pandemic.

## Mindfulness, Heartfulness and Well-being

Mindfulness is the awareness to be in a non-judgmental way in the present moment (Kabat-Zinn, 1994). The scientific research on the broad term “mindfulness” has increasingly grown in the last two decades (Van Dam et al., 2018). However, in many studies, poor methodology and unclarity in the definition of mindfulness has led to an exaggeration of the effects of mindfulness in some ways (Van Dam et al., 2018). Nevertheless, dispositional mindfulness, which describes mindfulness as a disposition or trait, can be regarded as the ability to act with an open and receptive mind, including self-regulation of attention and an open and non-judgmental orientation to experience (Bishop et al., 2004). Several measurements of mindfulness exist (e.g., FFMQ, Baer et al., 2008; FMI, Walach et al., 2006). The FFMQ, for example, includes the aspects of non-reactivity, observing, describing, acting with awareness, and non-judging.

Heartfulness, which is sometimes conceptualized as the warm side of mindfulness, can be investigated with the concepts of self-compassion (Neff, 2003), which is an indicator of heartfulness towards the own person, and gratitude (Emmons & McCullough, 2003), which is an indicator of heartfulness towards others (Voci et al., 2019). Self-compassion means compassion for oneself while suffering (Neff, 2003). The concept of self-compassion can be differentiated into the aspects of self-kindness vs. self-judgment, mindfulness vs. over-identification, and common humanity vs. isolation (Neff, 2003). Gratitude is the recognition of responding to others with grateful emotion due to their benevolence (McCullough et al., 2002). Research has shown that mindfulness and gratitude are positively related (Swickert et al., 2019).

Subjective well-being is a broad concept that can be differentiated into an affective and a cognitive component. The affective component can be understood as the emotional quality of one’s well-being. The cognitive aspect of well-being is measured mainly by the thoughts about one’s well-being (Diener et al., 1985). Among others, well-being can be measured with the Brief Inventory of Thriving (Su et al., 2014). Well-being can be differentiated between hedonic (subjective and emotional well-being) and eudaimonic well-being (Watermann, 1993). The hedonic approach focuses on happiness in a way that pleasure attainment and pain avoidance is important for well-being; the eudaimonic approach addresses meaning and self-realization and focuses on well-being as the degree to which a person is fully functioning (Ryan & Deci, 2001) Flourishing showed parallelism with high eudaimonic well-being, which includes components like meaning or purpose in life (Schotanus-Dijkstra et al., 2016). Flourishing means “to live within an optimal range of human functioning, one that connotes goodness, generativity, growth, and resilience” (Frederickson & Losada, 2005, p. 678).

## The Relation Between Mindfulness, Heartfulness, and Well-being

Several studies have investigated the relationship between mindfulness and well-being. For example, Josefsson et al., (2011) found an indirect effect of meditation experience on psychological well-being measured with the short version of Ryff’s Psychological Well-Being Scale (Ryff & Keyes, 1995). This effect has been mediated by the five mindfulness facets of the FFMQ (observing, non-reacting, acting with awareness, non-judging and describing while the mediation effect was the strongest for non-reacting. Moreover, mindfulness was related to well-being and performance in the workplace (Lomas et al., 2017). Several mediators in the relation between mindfulness and well-being have been found, such as emotional intelligence (Schutte & Malouf, 2011), resilience (Bajaji & Pande, 2016), and hope and optimism (Malinowski & Lim, 2015). Another relation could be found between dispositional mindfulness measured with the FFMQ and the concept of flourishing (Duan, 2016). However, Jon Kabat-Zinn (2004) already stated that mindfulness, besides its awareness qualities, also has a gentle emotional quality and can be described as heartfulness.

Regarding heartfulness, it has already been shown that self-compassion is one possible mechanism through which mindfulness is related to well-being (Hollis-Walker & Colosimo, 2011; Voci et al., 2019) examined the role of self-compassion and gratitude in relation to mindfulness, heartfulness, and psychological well-being. In their study, self-compassion mediated the relation between mindfulness and self-acceptance, autonomy, environmental mastery, and positive relations. Gratitude mediated the association between mindfulness and self-acceptance, environmental mastery, and positive relations. The authors concluded that mindfulness seems to foster higher levels of psychological well-being through heartfulness. Desai et al. (2022) used a specific heartfulness meditation practice (meditation practice bringing awareness to the heart and using the phone application called “heartsApp”). They demonstrated that heartfulness intervention significantly reduces the perceived stress score and the sleep quality index.

## The Goal of this Study

The study’s primary goal is to investigate the relationship between mindfulness, heartfulness, and well-being during the Coronavirus pandemic in higher education students. The stress may influence students’ well-being experienced during the pandemic. In general, it will be investigated how the perceived stress in the Coronavirus Pandemic, the aspects of heartfulness, mindfulness, gender, the strain of the Coronavirus Pandemic, and physical activity are related to well-being and flourishing. The following hypotheses will be tested:

1. In line with the findings of Voci et al., (2019), we assume that mindfulness predicts well-being (H1a) and flourishing (H1b) via mediating effects of self-compassion and gratitude (i.e., aspects of heartfulness). Furthermore, we investigate the five facets of mindfulness (non-reactivity, observing, describing, acting with awareness, non-judging) as possible predictors of well-being (H1.1a) and flourishing (H1.1b) via mediating effects of self-compassion and gratitude.
2. Students’ perceived stress during the Coronavirus Pandemic (Zurlo et al., 2020) harms their well-being (H2a) and flourishing (H2b).
3. Physical activity has a positive effect on students’ well-being (H3a) and flourishing (H3b) (Maher et al., 2021).
4. An exploratory analysis will investigate how the perceived stress in the Coronavirus Pandemic and gender contribute to well-being (H4a) and flourishing (H4b) under the consideration of heartfulness, mindfulness, gender, strain of the pandemic, and physical activity. Gender was considered a further predictor because female students showed more often depressive syndromes than male students during the Coronavirus Pandemic (Volken et al., 2021).

## Methods

### Participants

The sample consists of *N* = 216 students (124 women and 92 men) from various German universities. Participants’ age ranged from 18 to 31 (*M* = 21.96, *SD* = 1.99). Sixteen participants reported having been infected with Covid-19 in the past, 124 students reported an infection of a family member or a close friend, and 74 were already vaccinated. An a-priori power analysis with G*Power (Faul et al., 2007) showed that assuming small effects of *f*² = 0.10, a power of 0.80, and an alpha level of 0.05, a sample size of *N* = 185 is needed in multiple regression analyses including 12 predictor variables (see Statistical Analyses section).

The study was conducted according to the declaration of Helsinki. All participants were informed of the goal and the anonymity of the study, and the anonymity of the data storage. Furthermore, information was provided on the right to refuse to participate in the study or to withdraw consent to participate at any time without reprisal. All participants gave their informed consent before inclusion in the study in the online survey. The study was preregistered at https://osf.io/sb7t9/.

## Material

### Demographic Questionnaire

In the demographic questionnaire, the following questions were asked: “How old are you?”, “What is your gender?”, “Have you had a Covid-infection?” (*yes, no*), “Did one or more of your family members or close friends suffer from Covid?” (*yes, no*), “Have you already had a vaccination?” (*yes, no*), “How strained do you feel by the Coronavirus-Pandemic in your own life in general?” (5-point Likert scale ranging from 1 = *not at all* to 5 = *very much*).

### Well-being

**Brief Inventory of Thriving.** The Brief Inventory of Thriving (BIT; Su et al., 2014, German version: Hausler et al., 2017) assesses well-being with ten items which must be answered on a 5-point Likert scale ranging from 1 = *strongly disagree* to 5 = *strongly agree*. One of the items is: “My life has a clear sense of purpose.” Hausler et al., (2017) provided support for the reliability and validity of the German scale. The present study revealed a good internal consistency (Cronbach’s alpha = 0.81).

**Flourishing Scale.** The Flourishing Scale (FS; Diener et al., 2010, German version: Esch et al., 2013) is an eight-item questionnaire. One of the items is: “I lead a purposeful and meaningful life”: Participants rated each item on a 7-point Likert Scale, ranging from 1 = *strongly disagree* to 7 = *strongly agree*. Esch et al., (2013) supported the reliability and validity of the German scale, with Cronbach’s alphas ranging between 0.79 and 0.85. The present study also revealed a good internal consistency (Cronbach’s alpha = 0.85).

### Mindfulness

**Five-Facet Mindfulness Questionnaire**. The Five-Facet Mindfulness Questionnaire (FFMQ; Baer et al., 2008; German version: Michalak et al., 2016) is composed of five sub-dimensions, namely observing (e.g., “When I take a shower or bath, I stay alert to the sensations of water on my body”), non-reactivity (e.g., “I watch my feeling without getting lost in them”), acting with awareness (e.g., “I find my mind doing things without paying attention,” inverted), non-judging (e.g., “I disapprove of myself when I have irrational ideas,” inverted), and describing (e.g., “I can usually describe how I feel at the moment in considerable detail”). Each subscale includes seven to eight items, summing up to an overall item number of 39. Participants rated the items on a 5-point Likert scale, ranging from 1 = *applies very rarely* to 5 = *applies very often*. A study with 550 German college students found internal consistencies between 0.74 and 0.90 for the subdimensions (Michalak et al., 2016). The present study revealed good internal consistencies for the factors non-judging (Cronbach’s alpha = 0.91), describing (Cronbach’s alpha = 0.88), observing (Cronbach’s alpha = 0.80), acting with awareness (Cronbach’s alpha = 0.84), and non-reactivity (Cronbach’s alpha = 0.83). The reliability for the total score was Cronbach’s alpha = 0.90.

### Heartfulness

**Self-Compassion Scale.** The Self-Compassion Scale (SCS; Neff 2003; German version: Hupfeld & Ruffieux 2011), on the one hand, comprises the positive elements of self-kindness (e.g., “I’m kind to myself when I am experiencing suffering”), common humanity (e.g., “I try to see my failings as part of the human condition”) and mindfulness (e.g., “When I am feeling down I try to approach my feelings with curiosity and openness”). On the other hand, it comprises the negative aspects of self-judgment (e.g., “I can be a bit cold-hearted towards myself when I’m experiencing suffering”), isolation (e.g., “When I’m feeling down, I tend to feel like most other people are probably happier than I am”), and over-identification (e.g., “When something upsets me, I get carried away with my feelings”). Responses must be given on a scale from 1 = *rarely* to 5 = *almost always*. The negative items were reverse coded for the analysis. According to the recommendation of Coroiu et al., (2017), the positive and negative scales were separately used in the analysis. The present study revealed good internal consistencies for the positive (Cronbach’s alpha = 0.84) and the negative scale (Cronbach’s alpha = 0.89).

**Gratitude Questionnaire.** The original scale of the Gratitude Questionnaire (GQ-6; Mc Cullough et al. 2002, German version: Hudecek et al., 2010) consists of six items (e.g., “I have so much in life to be thankful for”), which must be rated on a 7-point Likert Scale, from 1 = *strongly disagree* to 7 = *strongly agree*. In a prior study, Cronbach’s alpha was 0.82 (Hudecek et al., 2010). The present study also revealed good internal consistency (Cronbach’s alpha = 0.80).

### Physical Activity

**International Physical Activity Questionnaire Short Form.** The International Physical Activity Questionnaire Short Form (IPAQ-SF; Research Committee, 2005) is a questionnaire for the registration of physical activity in the last seven days. It includes seven items, asking participants how many days in intensive (VIG) and moderate (MOD) physical activity and walking people they have spent and how many hours and minutes they have experienced with the specific intensity. The overall activity was measured in MET-minutes (metabolic equivalent of task) per week by the following weighted sum: Total MET-minutes/week = 4 * MOD (METs*min*days) + 8 * VIG (METs*min*days). To calculate the score for physical activity, MET minutes/week were standardized.

### Stress During the Coronavirus Pandemic

**Covid-19 Student Stress Questionnaire.** The Covid-19 Student Stress Questionnaire (CSSQ; Zurlo et al., 2020) is a 7-item stress questionnaire for students during the Coronavirus Pandemic. An example item is: “How do you perceive the risk of contagion during this period of the COVID-19 pandemic”. Answers to each item were given on a 5-point Likert scale from 1 = *not at all stressful* to 5 = *highly stressful* (5). A satisfactory internal consistency (Cronbach’s alpha = 0.71) was found for the Italian version. For the German version, the questionnaire was forward and backward translated. The present study revealed acceptable internal consistency (Cronbach’s alpha = 0.70).

## Procedure

Students were informed through a newsletter about the study. The online questionnaire was implemented using SoSci Survey (Leiner, 2019) and made available to the participants. First, all participants gave informed consent and reported their gender and age. Afterwards, they filled out the BIT (Hausler et al., 2017), FS (Esch et al., 2013), FFMQ (Michalak et al., 2016), SCS (Hupfeld & Ruffieux, 2011), GQ-6 (Hudecek et al., 2010), IPAQ-SF, and the CSSQ (Zurlo et al., 2020). Then, they answered the questions regarding the Coronavirus Pandemic (e.g., the strain of the Coronavirus Pandemic). They were then thanked for their participation. On average, participants spent 824.81 s (*SD* = 220.33) on the questionnaire.

### Statistical Analysis

We estimated a path model in MPlus 8 (Muthén et al., 2017). For model estimation, we used the MLR estimator, which is robust against violations of normality assumptions. We used the full information maximum likelihood (FIML) procedure to deal with missing values. This model-based approach to handling missing data is unbiased under the missing at random (MAR) assumption and retains statistical power as no observations are deleted. Due to these advantages, FIML is considered superior to traditional missing data treatment methods such as listwise deletion. Moreover, several studies have shown that FIML and multiple imputation tend to produce similar estimates, while FIML has the advantage that it is relatively easy to implement as no additional datasets must be generated (e.g., Baraldi & Enders 2010; Lee & Shi, 2021).

To test our hypotheses (except for H1.1a and H1.1b), we regressed well-being and flourishing on self-compassion (positive and negative subscale), gratitude, mindfulness, and all covariates (physical activity, strain, and stress due to the Coronavirus Pandemic, gender, age, Infection of self and others, and vaccination), and we regressed self-compassion and gratitude on mindfulness. Moreover, we compared the results of this model with the results of a model without the mediator variables (i.e., without self-compassion and gratitude). To test hypotheses H1.1a and H1.1b, we conducted similar analyses, but considered the different facets of mindfulness, rather than the total score of the mindfulness scale. In all models, we allowed correlations between all exogenous variables, between the residuals of the two subscales of self-compassion and gratitude, and between the residuals of well-being and flourishing. Data is available at https://mfr.osf.io/render?url=https%3 A%2 F%2Fosf.io%2Fzh5wk%2Fdownload.

## Results

For a first overview, means, standard deviations, and bivariate correlations of the study variables are reported in Table 1. The means of the perceived Coronavirus stress, *t*(215) = 3.56, *p* < .01, *d* = 0.24, and strain, *t*(215) = 7.55, *p* < .01, *d* = 0.53, significantly differ from the neutral of the answering scale (3) towards the stressful pole.

**Table 1:** Means, Standard Deviations, and Correlations of the Study Variables

	*M*	*SD*	Well-being	Flourishing	Mindfulness	SCS pos.	SCS neg.	Gratitude	CV Stress	CV Strain
Well-being	3.73	0.54								
Flourishing	5.53	0.75	0.82^**^							
Mindfulness	3.31	0.48	0.59^**^	0.66^**^						
SC pos.	3.11	0.58	0.44^**^	0.47^**^	0.60^**^					
SC neg.	3.00	0.74	− 0.51^**^	− 0.51^**^	− 0.66^**^	− 0.64^**^				
Gratitude	5.68	0.99	0.49^**^	0.47^**^	0.44^**^	0.34^**^	− 0.32^**^			
CV Stress	3.18	0.74	− 0.09	− 0.05	− 0.17^*^	− 0.14^*^	0.20^**^	− 0.02		
CV Strain	3.59	1.11	− 0.22^**^	− 0.11	− 0.19^**^	− 0.18^*^	0.36^**^	− 0.13	0.61^**^	
PA	4007.26	3434.54	0.19^**^	0.22^**^	0.17^*^	0.07	− 0.06	− 0.10	− 0.08	− 0.09

*Note*. SC = Self-Compassion, CV = Coronavirus, PA = Physical Activity. *p < .05,**p < .01

**Fig. 1:**
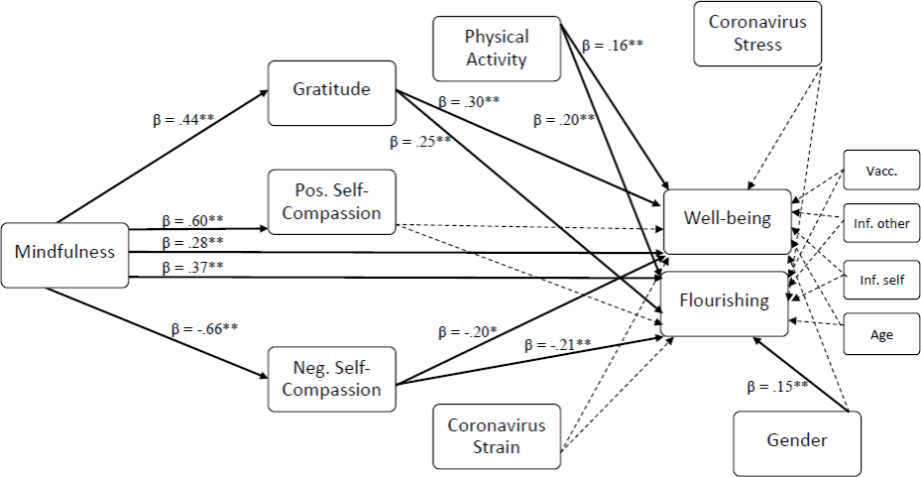
Model of the Predictors of Well-Being and Flourishing. Dotted lines show non-significant effects. Inf. Other = Infection of relatives or friends, Inf. Self = own Infection, Vacc. = already vaccinated. All βs and *CI*s are reported in Table 2. *p < .05, **p < .01

Without the mediators, mindfulness predicted well-being, β = 0.557, *p* < .001, 95% *CI* [0.481, 0.763] and flourishing, β = 0.623, *p* < .001, 95% *CI* [0.786, 1.155]. Figure 1; Table 2 depict the results of the path model including the global score of mindfulness. As shown, mindfulness predicted gratitude, β = 0.438, *p* < .001, 95% *CI* [0.328, 0.548], and self-compassion measured with both the positive scale, β = 0.603, *p* < .001, 95% *CI* [0.513, 0.692], and the negative scale, β = − 0.656, *p* < .001, 95% *CI* [-0.735, -0.578]. Furthermore, mindfulness predicted well-being directly, β = 0.284, *p* < .001, 95% *CI* [0.130, 0.438], and through mediating effects of gratitude, β = 0.300, *p* < .001, 95% *CI* [0.184, 0.415], and self-compassion measured with the negative scale, β = − 0.204, *p* < .01, 95% *CI* [-0.374, -0.035], which was in line with Hypothesis 1a. However, in contrast to Hypothesis 1a, mindfulness did not predict well-being through mediating effects of self-compassion measured with the positive scale, β = 0.015, *p* = .809, 95% *CI* [-0.109, 0.140]. Moreover, the perceived stress of the Coronavirus Pandemic did not predict well-being, which contradicted Hypothesis 2a, β = 0.035, *p* = .620, 95% *CI* [-0.104, 0.174]. However, in line with Hypothesis 3a, physical activity predicted well-being, β = 0.163, *p* < .001, 95% *CI* [0.051, 0.275]. Gender, β = 0.040, *p* = .479, 95% *CI* [-0.070, 0.150], and the strain of the Coronavirus Pandemic, β = − 0.067, *p* = .416, 95% *CI* [-0.230, 0.095], were no significant predictors of well-being (Hypothesis 4a). Mindfulness, physical activity, the Coronavirus stress, the strain of the Coronavirus Pandemic, gender, and all covariates explained 38.6% of the variance in well-being. Adding the aspects of heartfulness as mediators, 47.4% could be explained.

**Table 2:** Effects in the Main Analyses

	Well-being			Flourishing			SC pos.			SC neg.			Gratitude		
	β	*95% CI* *(LL)*	*95% CI* *(UL)*	β	*95% CI* *(LL)*	*95% CI* *(UL)*	β	*95% CI* *(LL)*	*95% CI* *(UL)*	β	*95% CI* *(LL)*	*95% CI* *(UL)*	β	*95% CI* *(LL)*	*95% CI* *(UL)*
Mindfulness	0.28	0.14	0.50	0.37	0.37	0.79	0.60	0.61	0.86	-0.66	-1.15	-0.88	0.44	0.63	1.18
SC pos.	0.02	-0.10	0.13	0.01	-0.15	0.17									
SC neg.	-0.20	-0.27	-0.03	-0.21	-0.37	-0.06									
Gratitude	0.30	0.10	0.22	0.25	0.11	0.26									
PA	0.16	0.03	0.14	0.20	0.06	0.23									
CV stress	0.04	-0.08	0.13	-0.01	-0.14	0.13									
CV strain	-0.07	-0.11	-0.05	0.08	-0.04	0.14									
Age	-0.06	-0.04	0.01	0.02	-0.03	0.04									
Gender	0.04	-0.08	0.16	0.15	0.08	0.38									
Infection self	0.06	-0.03	0.28	0.01	-0.24	0.32									
Infection other	0.01	-0.09	0.12	0.03	-0.09	0.19									
Vaccination	0.02	-0.09	0.13	0.05	-0.06	0.24									

*Note*. SC = Self-Compassion, CV = Coronavirus, PA = Physical Activity

In line with hypothesis 1b, mindfulness predicted flourishing directly, β = 0.373, *p* < .001, 95% *CI* [0.240, 0.506], and through mediating effects of self-compassion measured with the negative scale, β = − 0.209, *p* < .001, 95% *CI* [-0.359, -0.058], and gratitude, β = 0.249, *p* < .001, 95% *CI* [0.152, 0.346]. Mindfulness did not predict flourishing through self-compassion measured with the positive scale, β = 0.010, *p* = .869, 95% *CI* [-0.133, 0.134], which contradicted hypothesis 1b. Contrary to our hypothesis 2b, the perceived stress of the Coronavirus Pandemic did not predict flourishing, β = − 0.006, *p* = .929, 95% *CI* [-0.137, 0.125]. Physical activity predicted flourishing, β = 0.196, *p* < .001, 95% *CI* [0.076, 0.316] (Hypothesis 3b). Gender, β = 0.159, *p* < .001, 95% *CI* [0.053, 0.246], predicted flourishing but the strain of the Coronavirus Pandemic, β = 0.078, *p* = .259, 95% *CI* [-0.057, 0.212], was no significant predictor of flourishing (Hypothesis 4b). Mindfulness, physical activity, the Coronavirus stress, the strain of the Coronavirus Pandemic, gender, and all covariates explained 47.2% of the variance of flourishing. Adding the aspects of heartfulness as mediators, 54.1% could be explained.

For hypothesis H1.1a, non-judging, β = 0.201, observing, β = 0.109, and acting with awareness, β = 0.361, were significant predictors of wellbeing. Adding the aspects of heartfulness (Table 1), gratitude partly mediated the relation between acting with awareness and mindfulness and fully mediated the relation between mindfulness and non-judging and observing, respectively. All other covariates except for physical activity were no significant predictors of well-being. For flourishing (H1.1b), non-judging, β = 0.199, describing, β = 0.136, observing, β = 0.150, and acting with awareness, β = 0.370, were significant predictors. Adding the aspects of heartfulness (Table 3), gratitude and self-compassion measured with the negative scale partly mediated the relation between mindfulness and acting with awareness and describing and fully mediated the relation between mindfulness and non-judging and observing, respectively. All other covariates except for physical activity and gender were no significant predictors of flourishing. Without the mediators, 49.6% of the variance of flourishing and 41.8% of the variance of well-being were explained by all predictors, including the covariates. In the model with the aspects of heartfulness as mediators, an incremental 6.2% of the variance of flourishing respectively 7.7% of well-being was predicted by all variables.

**Table 3:** Effects in the Additional Analyses

	Well-being			Flourishing			SC pos.			SC neg.			Gratitude		
	β	*95% CI* *(LL)*	*95% CI* *(UL)*	β	*95% CI* *(LL)*	*95% CI* *(UL)*	β	*95% CI* *(LL)*	*95% CI* *(UL)*	β	*95% CI* *(LL)*	*95% CI* *(UL)*	β	*95% CI* *(LL)*	*95% CI* *(UL)*
Non-judging	0.11	-0.01	0.13	0.09	-0.02	0.17	0.21	0.05	0.20	-0.44	-0.42	-0.27	0.08	-0.08	0.25
Describing	0.05	-0.04	0.11	0.10	0.01	0.19	0.12	0.01	0.18	0.06	-0.16	0.04	0.10	-0.07	0.31
Observing	0.03	-0.05	0.10	0.09	-0.01	0.20	0.22	0.09	0.28	0.01	-0.08	0.10	0.18	0.07	0.44
Act aware	0.24	0.08	0.29	0.26	0.15	0.41	0.08	-0.03	0.17	-0.17	-0.29	-0.06	0.30	0.21	0.65
Non react	-0.01	-0.11	0.08	0.01	-0.13	0.14	0.39	0.22	0.44	-0.34	-0.48	-0.27	0.04	-0.15	0.25
SC pos.	0.07	-0.06	0.18	0.05	-0.09	0.22									
SC neg.	-0.16	-0.25	0.01	-0.20	-0.38	-0.04									
Gratitude	0.28	0.09	0.22	0.22	0.09	0.25									
PA	0.17	0.03	0.15	0.20	0.06	0.24									
CV stress	0.05	-0.07	0.14	0.01	-0.13	0.14									
CV strain	-0.06	-0.11	0.05	0.08	-0.04	0.14									
Age	-0.03	-0.04	0.02	0.03	-0.02	0.05									
Gender	0.01	-0.11	0.14	0.13	0.04	0.35									
Infection self	0.07	-0.01	0.31	0.02	-0.21	0.34									
Infection other	0.01	-0.10	0.11	0.03	-0.10	0.18									
Vaccination	-0.01	-0.11	0.10	0.04	-0.09	0.21									

*Note*. SC = Self-Compassion, CV = Coronavirus, PA = Physical Activity, non react = non-reactivity, act aware = acting with awareness

## Discussion

Our first hypothesis could partly be confirmed: Mindfulness, which had a positive effect on self-compassion and gratitude, predicted well-being and flourishing by the mediating effect of gratitude and the negative self-compassion scale. Going into more detail and looking at the five facets of mindfulness, only acting with awareness, beside the other predictors, gratitude, the negative aspect of self-compassion, and physical activity were significant predictors of well-being. All predictors explained 49.5% of the well-being variance in the mediating model (Hypothesis 1.1a). Similar results appeared for flourishing as a criterion. Gratitude and the negative self-compassion scale mediated the relation between mindfulness and flourishing. When the aspects of heartfulness were added as mediators, describing, and acting with awareness, gratitude and the negative scale of self-compassion as mediators and physical activity and gender as covariates were significant predictors of flourishing; other demographic variables and the strain and the stress due to the Coronavirus Pandemic did not predict well-being and flourishing (Hypotheses 2a and 2b). However, physical activity predicted well-being (Hypothesis 3a) and flourishing (Hypothesis 3b). Our fourth exploratory analysis revealed that the Coronavirus strain did not influence well-being and flourishing, and gender was a predictor only for flourishing.

## Mindfulness and its relation to well-being and flourishing

In line with prior research, we showed that mindfulness is positively related to well-being (Harrington et al., 2014; Josefsson et al., 2011) and flourishing (Duan, 2016). Experimental intervention studies have already confirmed these correlational results as, for example, the effects of mindfulness training programs on life satisfaction (Poulin et al., 2008) and well-being (Scheepers et al., 2020). Furthermore, quasi-experimental studies demonstrated higher psychological well-being of meditators compared to non-meditators (Josefsson et al., 2011).

For the five facets of mindfulness, non-judging, observing, and acting with awareness were significant predictors of well-being. Josefsson et al., (2011) found similar results for non-judging and acting with awareness. Participants who are more mindful in their daily life or have experience with meditation are more satisfied with their life and report higher well-being. This seems particularly applicable to people who are more observant and act with more awareness and less reactivity and judgment. Flourishing was also predicted by non-judging, observing, and acting with awareness, and additionally, the aspect of describing predicted flourishing.

On the one hand, this suggests that flourishing and well-being are overlapping constructs. Flourishing correlates to satisfaction with life, positive and negative feelings, and resilience (Yildirim & Belen, 2019), and Ryffs’ scales of well-being (autonomy, mastery, growth, relationships, purpose, and self-acceptance) (Diener et al., 2010). On the other hand, the results provide evidence that well-being and flourishing also have distinctive aspects. Describing, which predicted flourishing, refers to the ability to label our own experiences and to express them in words to ourselves and others. This can be seen with the results of Schotanus-Dijkstra et al., (2016), who showed that people with high values of flourishing also have high values of conscientiousness. The relevance of the describing aspect in flourishing gives a hint that flourishing emphasizes the “What” of well-being. The relevance of non-reactivity to thriving underlines the “How” of well-being.

## The relation between mindfulness, heartfulness, and well-being

Our results confirm the mediating role of heartfulness, at least for the negative scale of self-compassion and gratitude, on the relation between mindfulness and well-being. This is in line with the study of Voci et al., (2019). Comparable results were found in Canadian students (Hollis-Walker & Colosimo, 2011). In that study, well-being is first significantly predicted by all five facets of mindfulness. When the six subscales of self-compassion were added, one negative subscale of self-compassion (isolation) and two facets of mindfulness (describing and acting with awareness) significantly predicted well-being. Therefore, the relationship between mindfulness and well-being is partly mediated by self-compassion. The authors of a study on older adults suggest that self-compassion can have a protective influence on well-being (Allen et al., 2012). In undergraduate students, depression and anxiety were predicted by subscales of self-compassion and mindfulness but not by gender (Soysa & Wilcomb, 2015). In our study, the negative aspects of self-compassion negatively influenced well-being and flourishing while the positive aspects had no additional positive effect. Awareness of one’s shortcomings seems to influence well-being more than a loving attitude towards oneself.

Furthermore, the following studies investigated single aspects of heartfulness and well-being on the one hand and heartfulness and mindfulness on the other hand: Regarding the first relation, gratitude is positively related to well-being (Bono et al., 2020; Portocarrero et al., 2020) and flourishing and the negative aspect of self-compassion is negatively related to these constructs (Zessin et al., 2015; Muris et al., 2018) showed positive relations of the positive self-compassion score with reassuring thoughts and social support seeking and positive relations of the negative scale with anxiety or depression. Kroshus et al. (2021) suggested that increasing self-compassion in students could be a promising way to promote their well-being. Psychological well-being correlated with all subscales of self-compassion in adolescent students (Sun et al., 2016). Furthermore, gratitude strongly predicted well-being among students during the Coronavirus Pandemic (Bono et al., 2020). Concerning the second relation, we could show that mindfulness is positively related to self-compassion (Hwang et al., 2019) and gratitude (Jiang et al., 2020; Baer et al., 2012) found significant correlations between the six subscales of self-compassion and the five subscales of mindfulness, with only two non-significant exceptions.

Furthermore, all subscales of mindfulness and self-compassion were significantly related to well-being (Baer et al., 2016). In the present study, the positive scale of self-compassion did not predict well-being beyond gratitude, mindfulness, and the negative scale of self-compassion. Neff & Dahm (2015) argued that self-compassion and mindfulness have overlapping and distinctive areas and that both constructs are related to well-being. The scope of the mindfulness subscale of self-compassion is narrower than general mindfulness and focuses more on accepting negative thoughts rather than paying attention to all kinds of experiences.

## Well-being in the Coronavirus Pandemic in students

Our results demonstrated that physical activity is positively related to students’ well-being. It is in line with a meta-analysis providing evidence for positive effects of physical activity on life satisfaction and positive affect, but not on negative affect (Wiese et al., 2018). Another meta-analysis confirms these effects: Physical activity was associated with subjective well-being, positive affect, and cognitive well-being, but not negative affect (Buecker et al., 2020). These results seemed stable during the Coronavirus Pandemic: During this time, physical activity positively affected psychological health in young Italian adults (Maugeri et al., 2020). In the United Kingdom, physical activity was a strong positive predictor for subjects’ well-being outcomes, whereas Coronavirus fear negatively predicted well-being outcomes (Wright et al., 2021). Physical activity partly mediated the relation between Coronavirus fear and well-being outcomes. It supports our results: Physical activity predicts well-being and flourishing, but the Coronavirus stress or strain cannot explain incremental variance on the criteria. Hence, physical activity should be made possible for the population, especially in difficult times. People should be motivated to be active as much as possible. In Vietnamese adults, COVID-19 stress was negatively correlated with well-being and self-compassion but not with gratitude (Nguyen & Le, 2021). A multi-mediator model revealed a partial mediation in that both COVID-19 stress, and gratitude mediated the relation of self-compassion and well-being. Data was collected in August 2020. North American employees were asked eight times weekly about their work-loneliness, depression, and self-compassion starting in March 2020 at the pandemic’s beginning (Andel et al., 2021). A positive relation between work-loneliness and depression symptoms over time was attenuated for participants with higher scores on self-compassion. Hence, during the Coronavirus pandemic’s peak and its restrictions, gratitude and self-compassion could positively affect peoples’ well-being. Our results show similar relations of gratitude, self-compassion measured with the negative scale, and well-being, except for the Coronavirus Stress. It could be because the situation had already calmed when our data was collected. Participants could have gotten used to the situation or even been in a positive, optimistic mood because many restrictions were slowly removing.

The influence of gender on well-being and flourishing is inconsistent. In the present study, gender predicted flourishing but not well-being. Men reported higher scores in flourishing. Su et al., (2014) found gender differences in well-being, and women scored higher on flourishing scales than men (Lee et al., 2021). For life satisfaction, no gender differences were found in a meta-analysis (Batz-Barbarich et al., 2018). In a study with Turkish participants, the fear of COVID-19 mediated the relation between self-compassion and well-being (Deniz, 2021), and fear of COVID-19 was negatively correlated to well-being and self-compassion. For Italian participants during the lockdown in March and April 2020, mindfulness was the strongest predictor of psychological distress compared to demographic variables (gender, age, living with a lover) or even weeks in lockdown (Conversano et al., 2020). Our results show that mindfulness was a strong predictor of well-being, but the stress due to the Coronavirus Pandemic could not explain any incremental variance in well-being. A possible reason for this could be that in our study, physical activity was a significant predictor of well-being and flourishing.

## Limitations

Data collection of our study took place in May 2021. The stress due to the Coronavirus Pandemic may have already subsided. Even though many restrictions were still effective, people could have gotten used to them. Hence, the stress during the Coronavirus Pandemic did not influence participants’ well-being or flourishing. Moreover, the Covid-19 Student Stress Questionnaire consisted of only seven items that were translated into German. Although internal consistency was comparable to the original questionnaire, the German version is not validated. Besides, the concept of heartfulness lacks clarity. There are different points of view on the definition of heartfulness (e.g., van’t Westeinde & Patel 2022), and we have adapted the one from Voci et al., (2019). Furthermore, our data were collected in a cross-section design only once at the end of the third wave in Germany. The generalizability of these results also must be discussed regarding the country where data were retrieved and the participant group of students. Finally, it can be seen as a limitation of our study that we conducted our analyses using manifest variables. We did so because of the relatively large number of variables, compared to the sample size. The manifest approach did not allow us to account for measurement error in the examined constructs. However, our analyses indicated that these constructs were assessed with at least acceptable reliability.

## Conclusion

During the Coronavirus Pandemic, mindfulness positively predicted well-being and flourishing. This relationship was mediated by gratitude and the negative aspects of self-compassion. Physical activity but not the stress due to the Coronavirus Pandemic could additionally predict participants’ well-being and flourishing. Hence, a mindful treatment of oneself with elements of heartfulness and sufficient exercise might be a promising approach for life satisfaction during difficult times. Therefore, physical activity should be possible even during stay-at-home orders.

## References

[CR1] Allen AB, Goldwasser ER, Leary MR (2012). Self-compassion and well-being among older adults. Self and Identity.

[CR2] Andel SA, Shen W, Arvan ML (2021). Depending on your own kindness: The moderating role of self-compassion on the within-person consequences of work loneliness during the COVID-19 pandemic. Journal of Occupational Health Psychology.

[CR3] Baer RA, Smith GT, Lykins E, Button D, Krietemeyer J, Sauer S, Walsh E, Duggan D, Williams JMG (2008). Construct validity of the five-facet mindfulness questionnaire in meditating and nonmeditating samples. Assessment.

[CR4] Baer RA, Lykins EL, Peters JR (2012). Mindfulness and self-compassion as predictors of psychological well-being in long-term meditators and matched nonmeditators. The Journal of Positive Psychology.

[CR5] Bajaj, B., & Pande, N. (2016). Mediating role of resilience in the impact of mindfulness on life satisfaction and affect as indices of subjective well-being. *Personality and Individual Differences, 93, 63–67.*10.1016/j.paid.2015.09.005

[CR6] Baraldi AN, Enders CK (2010). An introduction to modern missing data analyses. Journal of School Psychology.

[CR7] Batz-Barbarich C, Tay L, Kuykendall L, Cheung HK (2018). A meta-analysis of gender differences in subjective well-being: estimating effect sizes and associations with gender inequality. Psychological Science.

[CR8] Bertrand L, Shaw KA, Ko J, Deprez D, Chilibeck PD, Zello GA (2021). The impact of the coronavirus disease 2019 (COVID-19) pandemic on university students’ dietary intake, physical activity, and sedentary behaviour. Applied Physiology Nutrition and Metabolism.

[CR9] Bishop SR, Lau M, Shapiro S, Carlson L, Anderson ND, Carmody J, Segal ZV, Abbey S, Speca M, Velting D, Devins G (2004). Mindfulness: a proposed operational definition. Clinical Psychology: Science and Practice.

[CR10] Bono, G., Reil, K., & Hescox, J. (2020). Stress and well-being in urban college students in the US during the COVID-19 pandemic: Can grit and gratitude help?International Journal of Wellbeing, *10*. 10.5502/ijw.v10i3.1331

[CR12] Buecker, S., Simacek, T., Ingwersen, B., Terwiel, S., & Simonsmeier, B. A. (2020). Physical activity and subjective well-being in healthy individuals: a meta-analytic review. *Health Psychology Review*, 1–19. 10.1080/17437199.2020.1760728 10.1080/17437199.2020.1760728 32452716

[CR13] Büssing A, Rodrigues Recchia D, Hein R, Dienberg T (2020). Perceived changes of specific attitudes, perceptions and behaviors during the Corona pandemic and their relation to well-being. Health and Quality of Life Outcomes.

[CR14] Conversano, C., Di Giuseppe, M., Miccoli, M., Ciacchini, R., Gemignani, A., & Orrù, G. (2020). Mindfulness, age and gender as protective factors against psychological distress during Covid-19 pandemic. *Frontiers in Psychology*, *11*, 1900. 10.3389/fpsyg.2020.01900 10.3389/fpsyg.2020.01900 PMC7516078 33013503

[CR15] Coroiu A, Kwakkenbos L, Moran C, Thombs B, Albani C (2017). Structural validation of the self-compassion scale with a German general population sample. PlosOne.

[CR16] Deniz ME (2021). Self-compassion, intolerance of uncertainty, fear of COVID-19, and well-being: A serial mediation investigation. Personality and Individual Differences.

[CR17] Desai K, Gupta P, Parikh P, Desai A (2022). Impact of virtual heartfulness meditation program on stress, quality of sleep, and psychological wellbeing during the covid-19 pandemic: A mixed- method study. International Journal of Environmental Research and Public Health.

[CR18] Diener E, Emmons RA, Larsen RJ, Griffin S (1985). The Satisfaction with Life Scale. Journal of Personality Assessment.

[CR19] Diener E, Wirtz D, William T, Kim-Prieto C, Dong-Won C, Oishi S, Biswas-Diener R (2010). New well-being measures: short scales to assess flourishing and positive and negative feelings. Social Indicators Research.

[CR20] Dodd RH, Dadaczynski K, Orkan O, McCaffery L, Pickles K (2021). Psychological well-being and academic experience of university students in Australia during Covid-19. International Journal of Environmental Research and Public Health.

[CR21] Duan W (2016). Mediation role of Individual strengths in dispositional mindfulness and mental health. Personality and Individual Differences.

[CR22] Esch T, Jose G, Gimpel C, von Scheidt C, Michalsen A (2013). Die Flourishing Scale (FS) von Diener et al. liegt jetzt in einer autorisierten deutschen Fassung (FS-D) vor: Einsatz einer Mind-Body-medizinischen Fragestellung. Forschung Komplementärmedizin.

[CR23] Faul F, Erdfelder E, Lang AG, Buchner A (2007). G*Power 3: A flexible statistical power analysis program for the social, behavioral, and biomedical sciences. Behavior Research Methods.

[CR24] Harrington R, Loffredo DA, Perz CA (2014). Dispositional mindfulness as a positive predictor of psychological well-being and the role of the private self-consciousness insight factor. Personality and Individual Differences.

[CR25] Hausler M, Huber A, Strecker C, Brenner, Höge T, Höfer S (2017). Validierung eines Fragebogens zur umfassenden Operationlisierung von Wohlbefinden. Diagnostica.

[CR26] Hollis-Walker L, Colosimo K (2011). Mindfulness, self-compassion, and happiness in non-meditators: a theoretical and empirical examination. Personality and Individual Differences.

[CR27] Hupfeld J, Ruffieux N (2011). Validation of a German version of the self-compassion scale (SCS-D). Zeitschrift für Klinische Psychologie und Psychotherapie.

[CR28] Hwang YS, Medvedev ON, Krägeloh C, Hand K, Noh JE, Singh NN (2019). The role of dispositional mindfulness and self-compassion in educator stress. Mindfulness.

[CR29] IPAQ Research Committee (2005). *International physical activity questionnaire.* In Guidelines for data processing and analysis of the international physical activity questionnaire (IPAQ)-Short and long forms. www.ipaq.ki.se. (German version)

[CR30] Jiang, Y., Ren, Y., Zhu, J., & You, J. (2020). Gratitude and hope relate to adolescent nonsuicidal self-injury: Mediation through self-compassion and family and school experiences. *Current Psychology*, 1–8. 10.1007/s12144-020-00624-4

[CR31] Josefsson T, Larsman P, Broberg AG, Lundh LG (2011). Self-reported mindfulness mediates the relation between meditation experience and psychological well-being. Mindfulness.

[CR32] Kabat-Zinn J (1994). Wherever you go, there you are.

[CR34] Kroshus E, Hawrilenko M, Browning A (2021). Stress, self-compassion, and well-being during the transition to college. Social Science & Medicine.

[CR35] Lee MT, Bialowolski P, Weziak-Bialowolska D, Mooney KD, Lerner PJ, McNeely E, VanderWeele TJ (2021). Self-assessed importance of domains of flourishing: Demographics and correlations with well-being. The Journal of Positive Psychology.

[CR36] Lee T, Shi D (2021). A comparison of full information maximum likelihood and multiple imputation in structural equation modeling with missing data. Psychological Methods.

[CR37] Leiner, D. J. (2019). SoSci Survey (Version 3.1.06) [Computer software]. Available at https://www.soscisurvey.de

[CR38] Lomas T, Medina JC, Rupprecht II, Hart S, Eiroa-Orosa FJ (2017). The impact of mindfulness on well-being and performance in the workplace: an inclusive systematic review of the empirical literature. European Journal of Work and Organizational Psychology.

[CR40] Maher, J. P., Hevel, D. J., Reifsteck, E. J., & Drollette, E. S. (2021). Physical activity is positively associated with college students’ positive affect regardless of stressful life events during the COVID-19 pandemic. *Psychology of Sport and Exercise*, *52*. 10.1016/j.psychsport.2020.101826 10.1016/j.psychsport.2020.101826 PMC7568511 33100905

[CR41] Malinowski P, Lim HJ (2015). Mindfulness at work: positive affect, hope, and optimism mediate the relationship between dispositional mindfulness, work engagement and well-being. Mindfulness.

[CR42] Maugeri G, Castrogiovanni P, Battaglia G, Pippi R, D’Agata V, Palma A, Musumeci G (2020). The impact of physical activity on psychological health during Covid-19 pandemic in Italy. Heliyon.

[CR43] McCullough ME, Emmons RA, Tsang JA (2002). The grateful disposition: a conceptual and empirical topography. Journal of Personality and Social Psychology.

[CR44] Michalak J, Zarbock G, Drews M, Otto D, Mertens D, Ströhle G, Schwinger M, Dahme B, Heidenreich T (2016). Erfassung von Achtsamkeit mit der deutschen Version des Five Facet Mindfulness Questionnaires (FFMQ-D) [Measuring mindfulness with the German version of the Five Facets Mindfulness Questionnaires (FFMQ-D)]. Zeitschrift Für Gesundheitspsychologie.

[CR45] Muris P, van den Broek M, Otgaar H, Oudenhoven I, Lennartz J (2018). Good and bad sides of self-compassion: a face validity check of the self-compassion scale and an investigation of its relations to coping and emotional symptoms in non-clinical adolescents. Journal of Child and Family Studies.

[CR46] Muthén, B. O., Muthén, L. K., & Asparouhov, T. (2017). *Regression and mediation analysis using Mplus*. Muthén & Muthén

[CR47] Neff K (2003). The development and validation of a scale to measure self-compassion. Self and Identity.

[CR48] Neff, K. D., & Dahm, K. A. (2015). Self-compassion: What it is, what it does, and how it relates to mindfulness. *Handbook of Mindfulness and Self-Regulation* (pp. 121–137). Springer

[CR49] Nguyen TM, Le GNH (2021). The influence of COVID-19 stress on psychological well-being among Vietnamese adults: The role of self-compassion and gratitude. Traumatology.

[CR50] Portocarrero FF, Gonzalez K, Ekema-Agbaw M (2020). A meta-analytic review of the relationship between dispositional gratitude and well-being. Personality and Individual Differences.

[CR51] Poulin PA, Mackenzie CS, Soloway G, Karayolas E (2008). Mindfulness training as an evidenced-based approach to reducing stress and promoting well-being among human services professionals. International Journal of Health Promotion and Education.

[CR53] Ryan RM, Deci EL (2001). On happiness and human potentials: A review of research on hedonic and eudaimonic well-being. Annual review of psychology.

[CR54] Ryff CD, Keyes CLM (1995). The structure of psychological well-being revisited. Journal of Personality and Social Psychology.

[CR55] Sanilevici M, Reuveni O, Lev-Ari S, Golland Y, Levit-Binnun N (2021). Mindfulness-based stress reduction increases mental wellbeing and emotion regulation during the first wave of the Covid-19 pandemic: A synchronous online intervention study. Frontiers in Psychology.

[CR56] Sharma PK, Kumra R (2022). Relationship between mindfulness, depression, anxiety and stress: Mediating role of self-efficacy. Personality and Individual Differences.

[CR57] Scheepers RA, Emke H, Epstein RM, Lombarts KM (2020). The impact of mindfulness-based interventions on doctors’ well-being and performance: A systematic review. Medical Education.

[CR58] Schotanus-Dijkstra M, Pieterse ME, Drossaert CH, Westerhof GJ, De Graaf R, Ten Have M, Bohlmeijer ET (2016). What factors are associated with flourishing? Results from a large representative national sample. Journal of Happiness Studies.

[CR59] Schutte NS, Malouff JM (2011). Emotional intelligence mediates the relationship between mindfulness and subjective well-being. Personality and Individual Differences.

[CR60] Soysa CK, Wilcomb CJ (2015). Mindfulness, self-compassion, self-efficacy, and gender as predictors of depression, anxiety, stress, and well-being. Mindfulness.

[CR61] Su R, Tay L, Diener E (2014). The development and validation of the Comprehensive Inventory of Thriving (CIT) and the Brief Inventory of Thriving (BIT). Applied Psychology: Health & Well-Being.

[CR62] Sun X, Chan DW, Chan LK (2016). Self-compassion and psychological well-being among adolescents in Hong Kong: Exploring gender differences. Personality and Individual Differences.

[CR63] Swickert R, Bailey E, Hittner J, Spector A, Benson-Townsend B, Silver NC (2019). The mediational roles of gratitude and perceived support in explaining the relationship between mindfulness and mood. Journal of Happiness Studies.

[CR64] Van Dam N, van Vugt MK, Vago DR, Schalzl L, Saron CD, Meyer DE (2018). Mind the hype: A critical evaluation and prescriptive agenda for research on mindfulness and meditation. Perspectives on Psychological Science.

[CR65] van’t Westeinde A, Patel KD (2022). Heartfulness meditation: A yogic and neuroscientific perspective. Frontiers in Psychology.

[CR66] Voci A, Veneziani CA, Fuochi G (2019). Relating mindfulness, heartfulness, and psychological well-being: the role of self-compassion and gratitude. Mindfulness.

[CR67] Volken, T., Zysset, A., Amendola, S., Klein Swormink, A., Huber, M., von Wyl, A., & Dratva, J. (2021). Depressive symptoms in Swiss university students during the covid-19 pandemic and their correlates. International Journal of Environmental Research and Public Health, *18*, 1458. https://doi.org/10.3390/ijerph18041458 10.3390/ijerph18041458 PMC7913894 33557193

[CR69] Waterman AS (1993). Two conceptions of happiness: Contrasts of personal expressiveness (eudaimonia) and hedonic enjoyment. Journal of Personality and Social Psychology.

[CR70] Wiese CW, Kuykendall L, Tay L (2018). Get active? A meta-analysis of leisure-time physical activity and subjective well-being. The Journal of Positive Psychology.

[CR71] Wilson OA, Holland KE, Elliott LD, Duffey M, Bopp M (2021). The impact of the COVID-19 Pandemic on US college students’ physical activity and mental health. Journal of Physical Activity and Health.

[CR72] Wright LJ, Williams SE, van Veldhuijzen JJ (2021). Physical activity protects against the negative impact of coronavirus fear on adolescent mental health and well-being during the COVID-19 Pandemic. Frontiers in Psychology.

[CR73] Yildirim M, Belen H (2019). The role of resilience in the relationships between externality of happiness and subjective well-being and flourishing: A structural equation model approach. Journal of Positive School Psychology.

[CR74] Zacher H, Rudolph CW (2021). Individual differences and changes in subjective well-being during the early stages of the COVID-19 pandemic. American Psychologist.

[CR75] Zessin U, Dickhäuser O, Garbade S (2015). The relationship between self-compassion and well-being: A meta‐analysis. Applied Psychology: Health and Well‐Being.

[CR77] Zurlo MC, Della Volta C, Vallone F (2020). Covid-19 student stress questionnaire: Development and validation of a questionnaire to evaluate students’ stressors related to the Coronavirus Pandemic. Frontiers in Psychology: Health Psychology.

